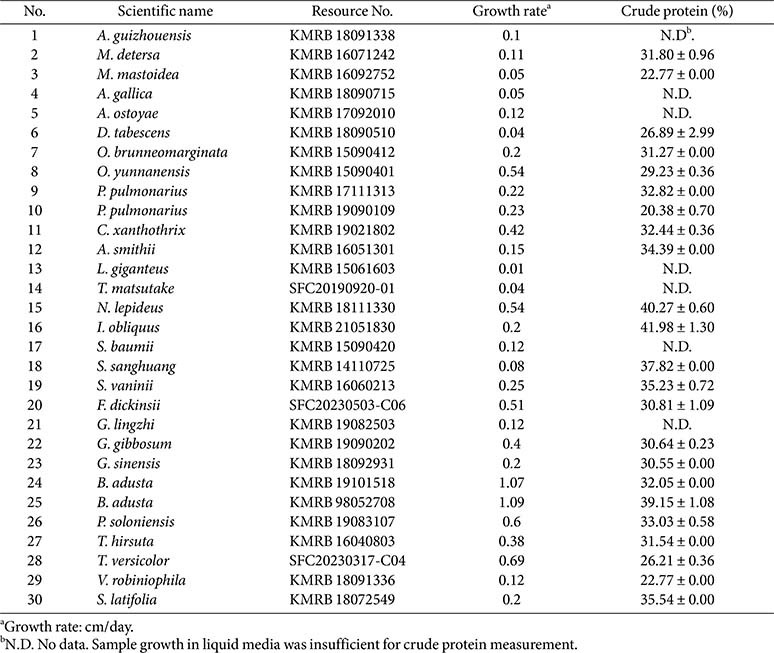# Integrated Assessment of Growth and Protein Content in Basidiomycetous Fungi for Mycoprotein Production

**DOI:** 10.4014/jmb.2510.10014

**Published:** 2025-11-26

**Authors:** Abel Severin Lupala, Yeon Ju Lee, Shinnam Yoo, Jiyun Choi, Jin Muk Lim, Su Bin Lee, Young Hoon Jung, Young Woon Lim

**Affiliations:** 1 School of Biological Sciences and Institute of Biodiversity, Seoul National University, Seoul 08826, Republic of Korea; 2 School of Food Science and Biotechnology, Kyungpook National University, Daegu 41566, Republic of Korea; 3 Department of Microbiology, Parasitology and Biotechnology, Sokoine University of Agriculture, P.O. Box 3019, Morogoro 67125, Tanzania

**Keywords:** Hyphal growth, crude protein, submerged fermentation, basidiomycetes, fungal biomass

## Abstract

The rising demand for sustainable and health-conscious protein sources has driven interest in fungal-derived mycoprotein as an alternative to conventional meat products. While commercial mycoprotein production predominantly relies on *Fusarium venenatum*, concerns over mycotoxin potential and limited strain diversity indicate the need to explore safer and edible basidiomycetes. In this study, 28 species across four taxonomic orders within Basidiomycota were screened for their potential as mycoprotein sources. Hyphal growth dynamics were measured on potato dextrose agar, and crude protein content was quantified from submerged mycelial cultures using the Kjeldahl method. Results revealed significant inter-order variation: Polyporales exhibited the fastest radial growth, while Agaricales grew the slowest. Highest crude protein levels were observed in *Inonotus obliquus* (41.98%), *Neolentinus lepideus* (40.27%), and *Bjerkandera adusta* (39.15%). The dual assessment of growth kinetics and nutritional value identified strains from Gloeophyllales, Hymenochaetales, and Polyporales as promising candidates for scalable mycoprotein development. These findings show the potential of basidiomycetous fungi as safe and effective sources of mycoprotein and provide a framework for future fermentation optimization and functional food innovation.

## Introduction

The global demand for sustainable and health-conscious protein sources is rapidly increasing, driven by population growth, environmental concerns, and the health risks associated with excessive red meat consumption [1]. In response, alternative protein sources, especially those that are environmentally sustainable and nutritionally valuable, have gained considerable attention [2]. Among these, mycoprotein, a protein derived from filamentous fungi, has emerged as a promising meat analogue due to its fibrous texture, high protein content, and potential health benefits [3].

The concept of microbial protein production, also known as single-cell protein (SCP), was initially proposed in the mid-20th century to address concerns about future protein shortages [4]. Early research focused on yeasts such as *Candida utilis*, *Saccharomyces cerevisiae*, and *Torula* species, which were widely used during wartime due to their nutritional value and scalable cultivation [5]. Subsequently, filamentous fungi such as *Aspergillus oryzae* and *Rhizopus oryzae* were investigated for their use in animal feed and food fermentation [6]. A breakthrough came in the 1960s with *Fusarium venenatum* A3/5 by Rank Hovis McDougall, which became the foundational organism for commercial mycoprotein production [7].

Currently, the majority of commercial mycoprotein production depends on *F. venenatum* [3, 8, 9]. While this species is classified by the FDA as generally recognized as safe, concerns have been raised about the long-term safety of *Fusarium* and *Aspergillus*-derived mycelia, particularly considering their potential to produce mycotoxins under certain conditions [10]. In contrast, many edible mushrooms belonging to the phylum Basidiomycota are widely consumed and have a long history of safe use [11], yet their potential for industrial-scale mycoprotein production remains underexplored.

Edible mushrooms offer several advantages as alternative protein sources. They can be cultivated on agricultural waste, require relatively little land or water, and have short production cycles compared to plant- or animal-based proteins [11, 12]. Species such as *Pleurotus ostreatus* can be harvested within 1–2 months and have demonstrated high protein content (19–35% dry weight), balanced amino acid profiles, and digestibility rates of 70–80% [13, 14]. Additionally, bioactive proteins isolated from edible mushrooms have shown antioxidant, immunomodulatory, and anticancer properties, expanding their potential applications in food and biotechnology [15].

Despite these advantages, studies on mushroom-derived mycoprotein have primarily focused on measuring total protein content or analyzing nutritional profiles [16-18]. Systematic efforts to screen and evaluate edible basidiomycetes for their mycoprotein production potential remain limited. In this pilot study, we aim to screen a broad range of basidiomycetous fungi by jointly assessing crude protein content and growth rate, thereby identifying strains capable of efficiently producing mycoprotein. Through this approach, we seek to explore safer and potentially more acceptable fungal sources of mycoprotein.

## Materials and Methods

### Fungal Strains Used in This Study

A total of 30 fungal strains were used in this study. They were classified into 28 species, 19 genera, 14 families, and four orders within the class Agaricomycetes (Table 1). All strains were isolated from fruiting bodies collected in forests throughout Korea and deposited in the Seoul National University Fungus Collection (SFC).

Genomic DNA was extracted from mycelial tissues of each fungal strain grown on potato dextrose agar (PDA)(using 5 mm diameter blocks) following the manufacturer’s instructions of the AccuPrep Genomic DNA extraction kit (Bioneer Co., Republic of Korea). Polymerase chain reaction (PCR) was conducted to amplify the internal transcribed spacer (ITS) region. Primer sets, amplification conditions, and subsequent analysis of PCR products and sequencing data were carried out as described in previous studies [19-21]. For species identification of the newly generated sequences, Basic Local Alignment Search Tool (BLAST) searches were performed against the GenBank database to identify the closest sequence matches. Strains exhibiting sequence identity of ≥ 97.5%were assigned to the corresponding species. All newly generated sequences were deposited in GenBank, and the corresponding accession numbers of strains used are listed in Table 1.

### Hyphal Growth Rate

The hyphal growth rate was measured as follows. A colony disc (diameter: 6.5 mm), obtained using a cork borer, was placed at the edge of a potato dextrose agar (PDA) plate with a diameter of 9.0 cm. The PDA plates were incubated at 25°C for 2 weeks, and the longest hyphal extension from the center of the disc was measured daily. Hyphal growth rate measurements were obtained in triplicate.

### Determination of Crude Protein Content

The fungal strains were pre-cultured on PDA plates at 25°C until the Petri dishes were fully colonized. Mycelial colonies were collected using a cork borer (diameter: 13 mm), and 15 colony discs were transferred into 150 ml of potato dextrose broth (PDB) and incubated at 25°C for 6 days at 150 rpm. After incubation, the fungal cultures were filtered through Miracloth (EMD Millipore Co., USA) to collect mycelial pellets. The collected pellets were washed with sterile distilled water and then lyophilized using a freeze dryer (FreeZone 2.5 L Benchtop, Labconco, USA). The dried mycelial pellets were ground into a fine powder, and 0.3 g from each sample was used for crude protein determination using the Kjeldahl method.

The Kjeldahl analysis was performed according to the AOAC [22]. In brief, 15 ml of 95% sulfuric acid (DUKSAN, Republic of Korea) and a K_2_SO_4_–Se catalyst tablet (KjTabs VST Kjeldahl Catalyst Tablets, VELP Scientifica Srl, Italy) were added to each sample, and digestion was carried out at 420°C for 1 h using a digestion unit (DK 20, VELP Scientifica Srl). The digested samples were then distilled using 30% NaOH and collected in a 4% boric acid solution using a semi-automatic distillation unit (UDK 139 Semi-automatic distillation unit, VELP Scientifica Srl). Finally, titration was performed using 0.1 M HCl to determine the nitrogen content. The crude protein content was calculated by multiplying the total nitrogen content by a conversion factor of 6.25.

### Statistical Analysis

Pearson’s product–moment correlation was performed to assess the linear relationship between hyphal growth rate (cm/day) and crude protein content (% dry weight). Analyses were conducted across all isolates and within each fungal order. For the overall dataset, correlations were computed using the cor.test function in R (v4.3.0). Order-specific correlations were obtained by grouping isolates according to taxonomic assignment and calculating Pearson correlation coefficients within each group.

## Results

### Hyphal Growth Dynamics by Taxonomic Groups

**Fig. 1:**
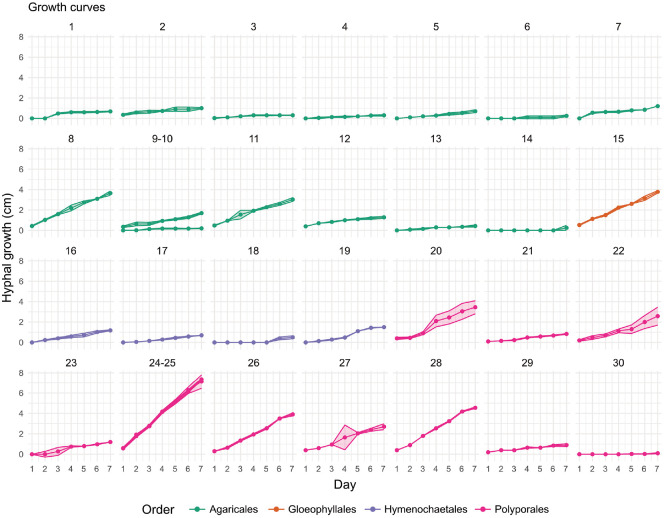
Hyphal growth dynamics of 28 basidiomycetous fungi measured over a 7-day incubation period on potato dextrose agar (PDA) at 25°C. Each subgraph represents an individual species, with numbers above corresponding to species numbers in Table 1. Line colors correspond to fungal orders. Curves represent mean radial growth (cm) per day, with standard deviations calculated from triplicate measurements.

The hyphal growth rates of 30 basidiomycetous strains were assessed over a 7-day incubation period (Fig. 1). Strains within the Polyporales exhibited the fastest average growth rate, approximately 0.42 cm/day. Among these, *Bjerkandera adusta* reached the edge of the Petri dish by day 6 and recorded a verified rate of 1.09 cm/day, the fastest in the dataset. Other Polyporales strains, including *Trametes versicolor* and *Fomitopsis dickinsii*, also demonstrated rapid and uniform radial expansion. Strains belonging to the genus *Ganoderma* exhibited slow to moderate hyphal extension, with colony diameters smaller than those of other Polyporales representatives. In the order Agaricales, the average radial growth was the lowest, around 0.163 cm/day, and most strains exhibited colony diameters of less than 3 cm by day 7. Species such as *Desarmillaria tabescens* and *Macrolepiota mastoidea* recorded particularly slow extension rates. *Oudemansiella yunnanensis*, a member of the Physalacriaceae within Agaricales, reached a verified growth rate of 0.54 cm/day, showing a deviation from the general trend observed in the order. The orders Hymenochaetales and Gloeophyllales exhibited moderate radial growth, with average rates of 0.23 cm/day and 0.57 cm/day, respectively. *Neolentinus lepideus*, the sole representative of Gloeophyllales in this study, displayed consistent and dense mycelial expansion under the same conditions.

### Crude Protein Content by Taxonomic Groups

The crude protein content among the tested filamentous fungi varied significantly, ranging from 20.38% to 41.98% (Table 2). For seven species, crude protein content could not be determined (N.D.) due to insufficient biomass production in liquid culture, which precluded Kjeldahl analysis. These cases included *Agaricus guizhouensis*, *Armillaria gallica*, *A. ostoyae*, *Ganoderma lingzhi*, *Leucopaxillus giganteus*, *Tricholoma matsutake*, and *Sanghuangporus baumii*.

Clear differences were observed among orders and families. Hymenochaetales showed the highest values, with *Inonotus obliquus* (KMRB 21051830; 41.98 ± 1.30%) and *Sanghuangporus sanghuang* (KMRB 14110725; 37.82 ± 0.00%) as top performers, resulting in a family average of 38.34 ± 3.41% for Hymenochaetaceae. Strains within Polyporales displayed a wide range of values, from 26.21% in *Trametes versicolor* to 39.15% in *B. adusta*. Other families within the order, including Piptoporellaceae (*Pseudophaeolus soloniensis*), Polyporaceae (*T. hirsuta*, *T. versicolor*, *Vanderbylia robiniophila*), and Sparassidaceae (*S. latifolia*), produced moderate to high values, while Ganodermataceae (*G. gibbosum*) and Grifolaceae (*G. sinensis*) showed intermediate levels.

Gloeophyllales, represented only by *Neolentinus lepideus*, reached 40.27 ± 0.60%. Agaricales showed the broadest variability, with moderate values in *Agrocybe smithii* (34.39 ± 0.00%), *Coprinellus xanthothrix* (32.44 ± 0.36%), and *Pleurotus pulmonarius* (32.82 ± 0.00%), but much lower values in *D. tabescens* (26.89 ± 2.99%) and *M. mastoidea* (22.77 ± 0.00%). Considerable strain-level variation within species was observed, as shown in *B. adusta* (32.05% vs 39.15%) and *P. pulmonarius* (32.82% vs 20.38%).

### Integrating Hyphal Growth and Protein Yield for Mycoprotein Potential

**Fig. 2:**
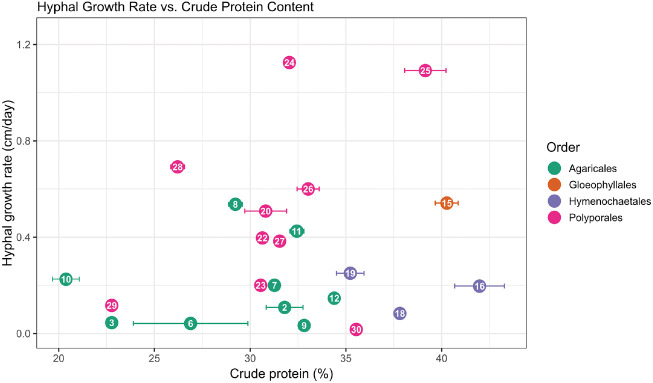
Integrated assessment of hyphal growth rate and crude protein content in 23 basidiomycetous fungi. The x-axis shows crude protein content (% dry weight) and the y-axis shows hyphal growth rate (cm/day). Species are grouped by order, represented by different circle colours. Numbers inside circles correspond to species numbers in Table 2. Each circle denotes the mean value per species, with error bars on the x-axis indicating the standard deviation of crude protein content.

Integrated evaluation of both hyphal growth and crude protein content revealed several high-performing strains. *I. obliquus* (41.98%), *N. lepideus* (40.27%), and *B. adusta* (39.15%) recorded elevated crude protein and exhibited favorable radial growth rates (Fig. 2). *O. yunnanensis* achieved a moderate crude protein level close to 30% and showed rapid mycelial expansion relative to other *Agaricales* strains.

Patterns across orders showed that Hymenochaetales and Gloeophyllales contained strains combining high protein content with moderate to rapid growth. In Polyporales, values ranged broadly, with *B. adusta* among the highest performers and *T. versicolor* among the lowest. Within this order, families such as Phanerochaetaceae (*B. adusta*) and Piptoporellaceae (*P. soloniensis*) showed profiles characterized by both adequate growth and intermediate to high protein yield. Across all taxa, integration of growth and protein measurements distinguished Gloeophyllales, Hymenochaetales, and Polyporales from the generally lower-performing Agaricales.

Pearson correlation across all taxa showed no significant relationship between hyphal growth rate and crude protein content (r = 0.227, *p* = 0.323). Order-specific analyses suggested positive associations in Polyporales (r = 0.418) and Agaricales (r = 0.320), whereas Hymenochaetales exhibited a weak negative correlation (r = –0.156). Correlation could not be calculated for Gloeophyllales, as only a single representative species was available.

## Discussion

This study evaluated 30 basidiomycetous fungal strains for their suitability as mycoprotein sources, using hyphal growth dynamics and crude protein content as dual performance indicators. Although preliminary in scope, the results demonstrate considerable inter- and intra-order variation, reflecting both taxonomic diversity and strain-specific physiological traits.

Crude protein analysis revealed several promising candidates. The Hymenochaetales, specifically *I. obliquus* and *S. sanghuang*, yielded the highest protein levels, exceeding 37%. These results are consistent with previous reports emphasizing the nutritional richness, medicinal use, and biosynthetic potential of these taxa [14, 23, 24]. Additionally, *B. adusta* and *N. lepideus*, from the Polyporales and Gloeophyllales, respectively, also demonstrated substantial protein yields. This aligns with prior studies reporting their nutritional and industrial relevance [25-28], highlighting their biotechnological value for mycoprotein production.

Among Agaricales, protein content was more variable. While *P. pulmonarius* and *A. smithii* reached protein content levels above 32%, other representatives, such as *D. tabescens* and *M. mastoidea*, remained below 27%. This variability may be linked to their ecological strategies, with many Agaricales allocating resources to reproductive structures or specialized decay mechanisms rather than rapid mycelial growth [29]. Furthermore, protein content in Agaricales is known to fluctuate with substrate composition and developmental stage; for example, nitrogen supplementation often enhances protein yield, while strain-specific differences can lead to divergent nutritional outcomes [30, 31]. This suggests that the nutritional potential of Agaricales may be underestimated if only single substrates or growth phases are considered.

Hyphal growth assays indicated that Polyporales generally exhibited the most vigorous expansion, while Agaricales tended to show slower growth. However, strain-level differences were substantial, such as the relatively fast growth of *O. yunnanensis* within Agaricales. Since optimal growth conditions vary considerably among species, including factors such as nutrient composition, pH, and temperature [32-35], it is difficult to conclude that any particular family or order inherently possesses the fastest growth rate. Nonetheless, under the standardized culture conditions applied in this study, hyphal growth generally followed order-level patterns, but strain-level differences were clearly evident.

When both traits were considered together, several strains emerged as promising candidates. *B. adusta*, *I. obliquus*, and *N. lepideus* combined relatively rapid mycelial proliferation with protein levels comparable to *F. venenatum*, the benchmark species for commercial mycoprotein [9]. Such dual performance is advantageous for industrial scale-up, as it supports both nutritional quality and efficient biomass yield. While species from the Agaricales generally exhibited slower radial growth and moderate protein levels, exceptional cases indicate that targeted strain selection within traditionally low-yielding groups could still yield industrially viable candidates. At the same time, this order is widely cultivated for its nutritional value [36], and many species provide substantial amounts of mycelial biomass. These features underscore the importance of continued exploration of Agaricales as potential sources of mycoprotein.

Furthermore, this study broadens the scope of mycoprotein research, which has traditionally focused on filamentous fungi [37]. The inclusion of submerged mycelial biomass from basidiomycetous fungi provides a new perspective for sustainable protein production. Given their adaptability, enzymatic versatility, and long-standing recognition as edible mushrooms [11], they represent a valuable but underutilized resource in microbial protein biotechnology. Ultimately, this integrated approach combining hyphal growth analysis with crude protein quantification offers a robust and scalable screening strategy for identifying fungal strains suitable for mycoprotein applications. The present study supports that among basidiomycetous fungi, species belonging to the Gloeophyllales, Hymenochaetales, and Polyporales possess significant potential as promising sources for next-generation protein foods and functional ingredients.

While these results are encouraging, several limitations must be acknowledged. First, crude protein content was measured without amino acid profiling or digestibility assessment, which are essential for determining nutritional quality. Second, growth was assessed under standardized laboratory conditions, which may not fully reflect performance in a large-scale submerged fermentation system. Third, safety validation, including mycotoxin screening, was not performed. However, it is important to note that Basidiomycota species, including *I. obliquus*, *N. lepideus*, and *B. adusta*, are not known to produce mycotoxins. Mycotoxin production is largely restricted to filamentous Ascomycota (*e.g.*, *Aspergillus*, *Penicillium*, *Fusarium*) [38], whereas Basidiomycota generally produce non-mycotoxigenic secondary metabolites or, in certain toxic species, mushroom-specific toxins typically confined to fruiting bodies [39]. The key taxa identified here comprise edible or medicinal species (*I. obliquus*, *N. lepideus*) and non-edible yet non-toxic, biotechnologically important species (*B. adusta*), collectively supporting their safety as potential mycoprotein candidates [24-28]. Despite these limitations, the integrated screening approach adopted here provides a useful framework for identifying promising strains for further study.

## Conclusion

This screening demonstrated marked variation in protein yield and growth among basidiomycetous fungi. The highest-yielding species were found in Gloeophyllales and Hymenochaetales, while Polyporales showed a broad range of values from low to high, and Agaricales exhibited the greatest variability. Notably, *B. adusta*, *I. obliquus*, and *N. lepideus* combined high protein content with favorable growth, suggesting their potential as next-generation mycoprotein sources. Although limited in scale and analytical depth, the study establishes a foundation for future work by demonstrating that edible basidiomycetes can rival or complement *F. venenatum* in protein potential. By framing growth and protein yield as dual performance indicators, this study provides a scalable screening strategy for evaluating fungal strains in the context of sustainable protein development. Building on this framework, subsequent research should incorporate nutritional profiling, digestibility testing, and functional property evaluation to validate the industrial feasibility of basidiomycetous fungi as alternative protein sources.

## Figures and Tables

**Table 1:** List of basidiomycete species and ITS Accession numbers used in this study.

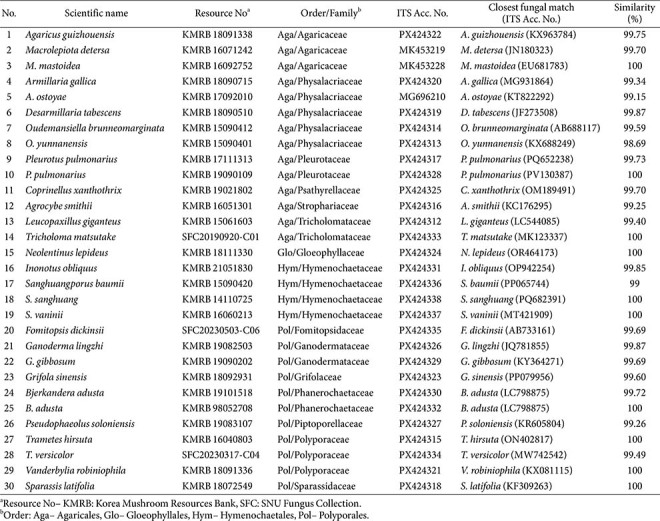

**Table 2:** Crude protein content and hyphal growth rate of different basidiomycetous fungi used in this study.